# *In Vivo* Degradation Behavior of the Magnesium Alloy LANd442 in Rabbit Tibiae

**DOI:** 10.3390/ma4122197

**Published:** 2011-12-15

**Authors:** Berit Ullmann, Janin Reifenrath, Dina Dziuba, Jan-Marten Seitz, Dirk Bormann, Andrea Meyer-Lindenberg

**Affiliations:** 1 Small Animal Clinic, University of Veterinary Medicine Hanover Foundation, Buenteweg 9, Hanover 30559, Germany; E-Mails: janin.reifenrath@tiho-hannover.de (J.R.); dina.dziuba@hannover-re.com (D.D.); 2 Hannover Centre for Production Technology, Institute of Materials Science, Leibniz University of Hanover, An der Universitaet 2, Garbsen 30823, Germany; E-Mails: seitz@iw.uni-hannover.de (J.-M.S.); dirk.bormann@trimet.de (D.B.); 3 Clinic for Small Animal Surgery and Reproduction, Centre of Clinical Veterinary Medicine, Faculty of Veterinary Medicine, Ludwig-Maximilians-Universitaet Muenchen, Veterinaerstr. 13, Munich 80539, Germany; E-Mail: andrea.meyer-lindenberg@chir.vetmed.uni-muenchen.de

**Keywords:** biodegradation, magnesium alloy, mechanical stability, animal model, µ-computed tomography

## Abstract

In former studies the magnesium alloy LAE442 showed promising *in vivo* degradation behavior and biocompatibility. However, reproducibility might be enhanced by replacement of the rare earth composition metal “E” by only a single rare earth element. Therefore, it was the aim of this study to examine whether the substitution of “E” by neodymium (“Nd”) had an influence on the *in vivo* degradation rate. LANd442 implants were inserted into rabbit tibiae and rabbits were euthanized after 4, 8, 13 and 26 weeks postoperatively. *In vivo* µCT was performed to evaluate the *in vivo* implant degradation behaviour by calculation of implant volume, density true 3-D thickness and corrosion rates. Additionally, weight loss, type of corrosion and mechanical stability were appraised by SEM/EDS-analysis and three-point bending tests. Implant volume, density and true 3-D thickness decreased over time, whereas the variance of the maximum diameters within an implant as well as the corrosion rate and weight loss increased. SEM examination revealed mainly pitting corrosion after 26 weeks. The maximum bending forces decreased over time. In comparison to LAE442, the new alloy showed a slower, but more uneven degradation behavior and less mechanical stability. To summarize, LANd442 appeared suitable for low weight bearing bones but is inferior to LAE442 regarding its degradation morphology and strength.

## 1. Introduction 

Implant materials must exhibit adequate strength, good biocompatibility as well as a reliable quality for their applications in osteosynthesis. 

Hitherto, non-resorbable titanium and its alloys as well as surgical steel and alloys based on cobalt-chromium were employed particularly for loaded bone [1,2]. Owing to undesirable effects, such as the so-called “stress shielding”, foreign-body reactions, mutagenicity and inflammations caused by the release of toxic metal ions, these materials must frequently be removed in a second procedure [2,3,4,5]. This represents an additional anaesthetic risk and leads to further expenses for the health system. In order to avoid these disadvantages, current research focuses on resorbable materials for osteosynthesis [2,3,6,7,8,9].

Polymers, such as PGA and PLA, as well as ceramics, such as β-TCP, or hydroxylapatite are employed as degradable materials [3,9,10]. Since these materials do not possess sufficient strength for weight bearing bones, they are mainly used in non-loaded bone structures [9,10]. In contrast to this, magnesium alloys have very similar mechanical characteristic values to bone [2,11] and magnesium is, moreover, an essential mineral for the body [11,12]. However, early studies carried out at the beginning of the 20th century using magnesium as an implant material demonstrated copious gas production due to a too rapid degradation of the magnesium material [13,14,15]. This lead to insufficient biocompatibility and, furthermore, to a premature loss in strength [16,17]. Further research using magnesium as a material for osteosynthesis played a minor role, presumably because of the development of stainless steel, and later titanium, as a medical product [8].

Currently, modern technology permits the light metal magnesium to be alloyed with various other elements such as, for instance, calcium, lithium, aluminum or the rare earths [2,7,8,18,19] in order to elevate the strength of magnesium and to reduce the corrosion rate [20].

For example, the magnesium alloy LAE442 which contains rare earth metals demonstrated good *in vivo* biocompatibility and homogeneous degradation behavior [6,7,8,21]. However, it is disadvantageous that the rare earths consist of a mixture of 15 individual elements belonging to the lanthanides plus scandium and yttrium [22] which, depending on the supplier, can be composed of different elemental proportions [8]. Thus, it is almost impossible to exactly predict the percentages of the individual elements. Adding just one individual element of the rare earths to the alloy would make the alloying reproducible and contribute to controlling the quality [23]. The main rare earth elements in the LAE442 alloy are cerium, lanthanum, neodymium and praseodymium [8,22]. For this reason, cerium was added as an individual element to the alloy (LACer442) in an investigation by Reifenrath *et al.* [23]. However, the alloy exhibited very poor *in vivo* biocompatibility [23].For the single element praseodymium, hepatotoxic effects in rats could be seen after intravenous administration [24]. Moreover, in contrast to neodymium, toxic effects were demonstrated in *in vitro* studies with cerium and lanthanum [25]. However, the results of *in vivo* studies, using lanthanum and neodymium as individual elements, are not available in the accessible literature. In one *in vitro* study using human bone cells, neodymium was classified as a suitable alloying element for biomedical applications [25]. Furthermore, used as an alloying element, neodymium leads to grain refining in magnesium compounds and therefore to increased strength and ductility [18,26,27]. Therefore, in the current study, it is to be tested whether the degradation behavior of the new LANd442 alloy, where the rare earth mixture has been replaced by the individual element neodymium, is comparable to the original LAE442 alloy. 

## 2. Results and Discussion

### 2.1. Clinical Investigations

The clinical investigation resulted in a low level of redness in the region of the surgical wound for a few days after the procedure. Six of the 18 animals showed a low level of subcutical emphysema for 1 to 4 days. One animal exhibited one-sided lameness for 74 days which, according to its severity, was treated with Meloxicam (0.15 mg/kg, Metacam^®^, Boehringer Ingelheim, Ingelheim, Germany) and buprenorphine hydrochloride (0.05 mg/kg, Bayer, Bayer AG, Leverkusen, Germany). Two other animals demonstrated a low level of one-sided lameness for one day (the 34th and 53rd days). 

### 2.2. Radiological Investigations

It was possible to radiologically establish that, with the exception of one pin, all were correctly located in the middle third of the tibia. This one implant from the 8 weeks groups was situated in the proximal third of the tibia but, in the course of the investigations, did not differ from the other implants in the same duration group. 

**Figure 1:**
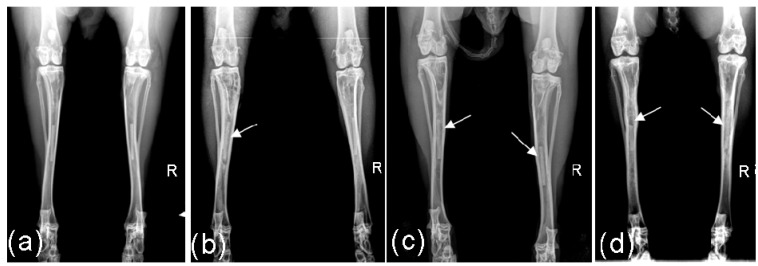
Radiological images of implants: (**a**) after 4 weeks implantation period: Score left 0, right 0; (**b**) after 8 weeks implantation period: Score left 1, right 0; (**c**) after 13 weeks implantation period: Score left 1, right 1; (**d**) after 26 weeks: Score 3 and low level of gas formation left, score 2 right.

In the 4 weeks groups no implants demonstrated radiological changes. In the other three groups, three of the 26 implants were assessed with a score of 1 after 8 weeks. After 13 weeks, 8 of the remaining 16 implants obtained the score 1 and one implant the score 2. Following 26 weeks implantation, five of the remaining ten implants were assessed with the score 1, two with score 2 and two with the score 3 (Figure 1). During the entire 26 weeks monitoring time, only one implant remained radiologically unchanged.

In only two implants during the 8th and the 26th week, respectively, a low level of gas development could be detected. 

The computed median was 0 over the entire time duration for the 4 and 8 weeks groups. The median attained a value of 1 in the 12th and 13th week for the 13 weeks groups. In the 26 weeks groups, the median value remained 1 from the 14th week to the end of the observation period. 

### 2.3. µ-Computed Tomographic Investigation of the *in Vivo* LANd442-Pins

**Figure 2:**
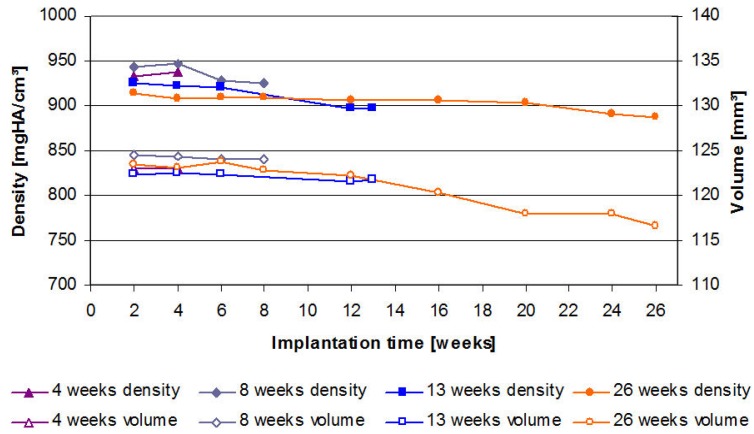
µ-computed tomographically educible reductions of implant volumes (open symbols) and densities (full symbols) of the LANd442-implants introduced into the rabbits’ tibiae plotted against the test period of up to 26 weeks.

For the 4 weeks groups, the volume of the implanted LANd442-pins remained relatively constant (123.0 mm^3^ to 122.9 mm^3^). From the 8th week, it was possible to observe a continuous low loss of volume (Figure 2) which, after 13 weeks, amounted to 0.6% (from 122.4 mm^3^ (SD 2.1) to 121.7 mm^3^ (SD 2.7)) compared to the initial scan. After 26 weeks, it was possible to determine a loss of volume of 5.5% (from 123.4 mm^3^ (SD 2.93) to 116.6 mm^3^ (SD 15.05)). One implant from the 26 weeks groups could not be evaluated owing to a loss of data. Neither significant differences between the groups, nor over the course of time occurred.

The density’s evaluation resulted in a low but significant (p = 0.03) increase in density for the 4 weeks groups (n = 10) of 0.5% from 933 mgHA/ccm (SD 82) to 937 mgHA/ccm (SD 84) from week 2 to week 4 (Figure 2). For the 8 weeks groups (n = 10), it was also possible to observe a low level of elevated density of 0.3% from the 2nd to the 4th week. This was, however, not significant (p = 0.104). The remaining two groups (13 weeks (n = 6) and 26 weeks (n = 9)) demonstrated mainly continuous density losses during the implant period. At the end of the respective observation periods of the 8, 13 and 26 weeks groups, a significant density loss occurred in comparison to the corresponding initial scans. These losses amounted to 2% (p = 0.028), 3% (p = 0.028) and again 3% (p = 0.008) for the 8, 13 and 26 weeks groups, respectively (Figure 2). It was not possible to establish significant differences between the groups at the end of the respective tests. 

A continuous loss of the 3D-thickness was exhibited. From the 12th week, a significant reduction (p = 0.007) of the 3D-thickness of 3% to 2.24 mm (SD 0.08) was demonstrated in comparison to the initial value (2.31 mm (SD 0.03)). This reduction attained a considerable loss (p = 0.003) of 11.69% (2.31 mm (SD 0.03) to 2.04 mm (SD 0.22)). It was possible to observe a significant (p = 0.042) difference between the 4 and the 26 weeks groups. A comparison of the remaining groups resulted in no significant differences. 

**Figure 3:**
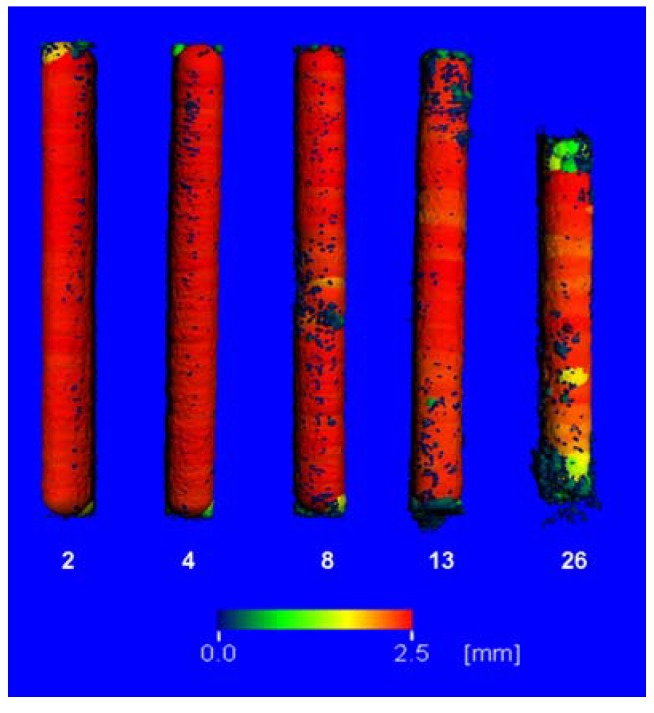
Images of the LANd442 implants’ true 3D-thicknesses following different implantation durations in the rabbits’ tibiae: from left to right: after 2 weeks, 4 weeks, 8 weeks, 13 weeks and after 26 weeks implantation periods. The colors correspond to the maximum diameters of the inserted balls; from red, yellow, green to blue, the diameter becomes smaller (red = 2.5 mm; blue = almost 0).

The mean 3D-thickness (Figure 3) did not change in the 4 weeks groups. 

**Figure 4:**
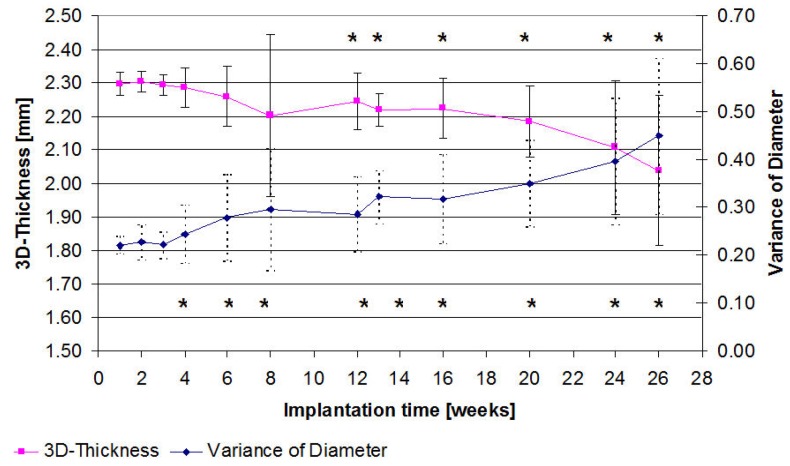
Representation of the 3D-thickness development and the variance of the implant’s diameter determined by µ-computed tomography plotted over the course of 26 weeks. Significant changes (p ≤ 0.05) compared to the second post implantation week are identified by a *.

The diameter’s variance mainly increased continuously from the initial scan (Figure 4). Across all the time groups, it was possible to observe significant (p = 0.032) elevations from the 4th week in comparison to each of the initial scans. The mean diameter’s variance was 195.7% of its initial value (0.23 to 0.45) after 26 weeks. It was possible to establish a significant difference (p = 0.035) between the final values of the 4 and 8 weeks groups but not between the 8 and 13 weeks or the 13 and 26 weeks groups. This was due to higher standard deviations.

The computed corrosion rates for LANd442, based on 4, 8 and 12 weeks, were 0.01 mm/y, 0.02 mm/y and 0.03 mm/y, respectively. It was possible to determine a value of 0.072 mm/y after 26 weeks. These rising values demonstrate an increasing corrosion rate towards the end of the implantation period.

### 2.4. X-Ray Diffractometry Following Explantation 

As an example, the surface of an implant, which exhibited a markedly white layer after explantation, was investigated with respect to its crystal structure. It was possible to verify the predominant presence of brucite (magnesium hydroxide). In addition to this, less classifiable hydroxides and oxide-hydroxide mixtures of the other alloying constituents; aluminum, lithium and neodymium, were found.

### 2.5. Investigation of the Pins Using Stereomicroscopy Following Explantation

**Figure 5:**
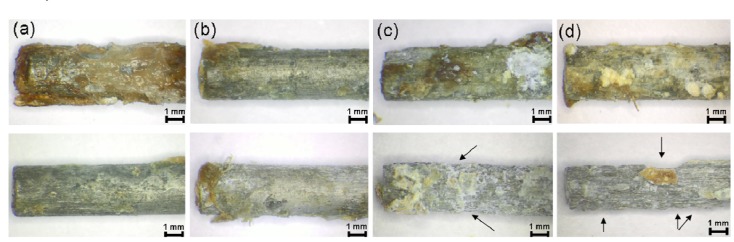
Stereomicroscopy images of explanted pins (×2 magnification): (**a**) 4 weeks; (**b**) 8 weeks; (**c**) 13 weeks; (**d**) 26 weeks; each prior to (top) and following (bottom) the hydrofluoric acid treatment: The arrows indicate the more severe corrosion.

Subsequent to explantation, it was possible to observe a brown-red mass on the implants, which almost covered the entire surface. In addition to this, white crystalline-like regions were found on the surface (Figure 5). These were most pronounced in the 13 and 26 weeks groups. All the pins exhibited an almost cylindrical shape after explantation. One implant from the 26 weeks groups exhibited eroded ends, significant corrosion pitting and a shorter length in comparison to the other implants from the same group.

After the treatment in hydrofluoric acid, bright silvery surfaces possessing longitudinal grooves were discernible on all the implants. These were least formed in the 4 weeks groups. The corrosion increased with increasing implantation duration. One implant from the 8 weeks groups showed a rounded end and was more severely corroded than the remaining implants after 8 weeks. However, this did not involve the implant which was radiologically more proximally situated in the tibia. The implants of the 13 weeks groups demonstrated, on average, a more severe corrosion than the implants in the 8 week group. It was possible to discern few, very shallow cavities after 13 weeks. 

In comparison to the other groups, the implants in the 26 weeks groups demonstrated the most severe corrosion. They exhibited a rough and uneven surface possessing longitudinal grooves as well as the previously described white, crystalline regions. The implants’ ends showed a tapered form and several shallow cavities on the surface (Figure 5(d) below).

### 2.6. SEM Investigation and the EDX Analysis

**Figure 6:**
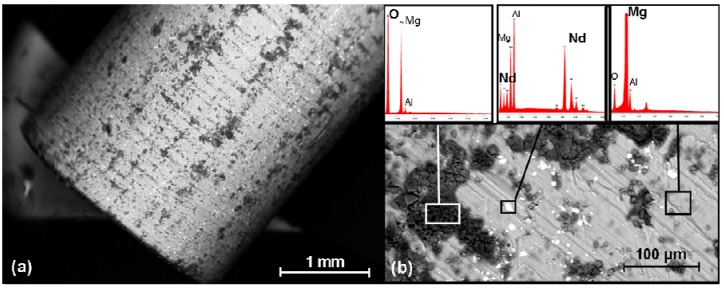
SEM images prior to implantation: (**a**) Overview of the pin’s surface prior to the test; (**b**) EDX analysis of the pin’s surface: preferentially detected Mg, Al, Nd and O. Each of the boxes mark the regions selected for the EDX analysis.

In the SEM, the pins showed a relatively smooth surface prior to implanting (Figure 6(a)), only the grooves due to manufacturing were discernible. In the EDX analysis, it was possible to identify oxygen and neodymium rich areas as well as magnesium and aluminium (Figure 6(b)).

**Figure 7:**
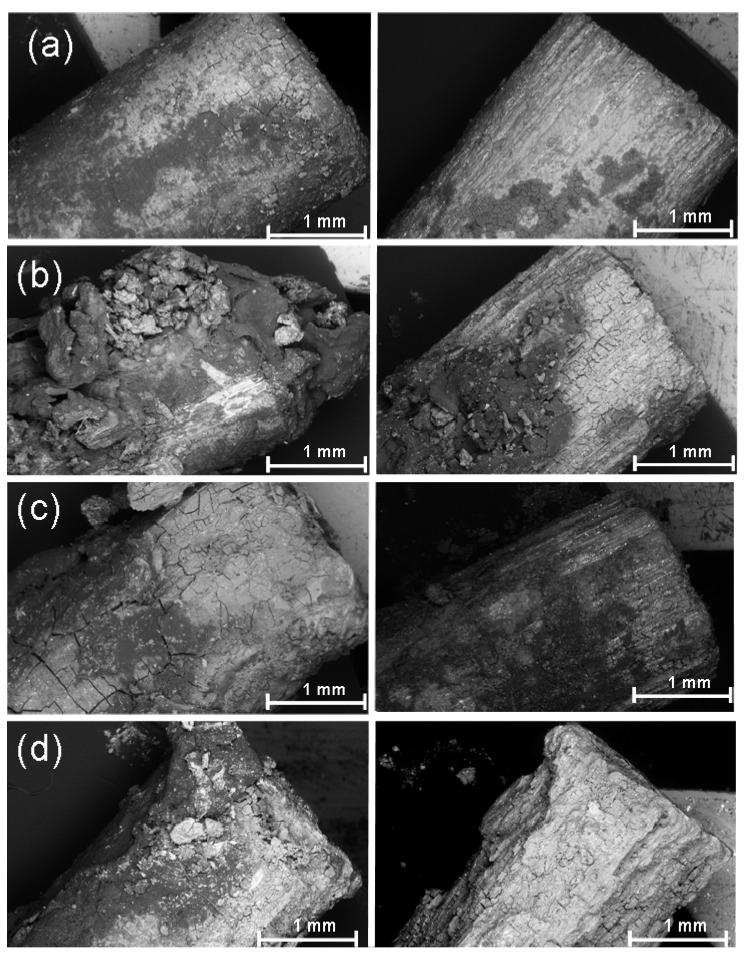
SEM images of the pin ends: (**a**) 4 weeks; (**b**) 8 weeks; (**c**) 13 weeks; (**d**) 26 weeks; each prior to (left) and following (right) hydrofluoric acid treatment

**Figure 8:**
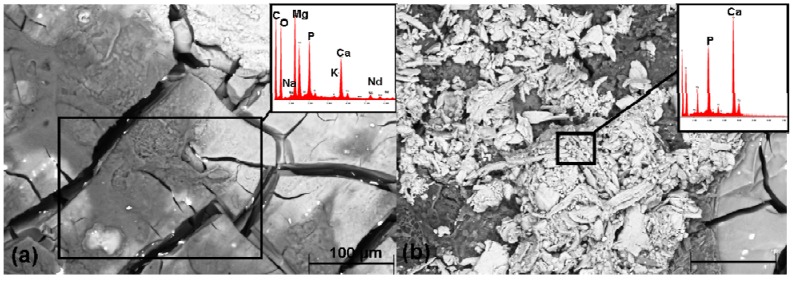
SEM images of the pin’s surface using a pin following 26 weeks implantation prior to the hydrofluoric acid treatment and the EDX analysis of the individual elements in the marked regions: (**a**) clump-like structures; (**b**) crystalline structures, verification of Ca and P on the pin’s surface. Each of the boxes mark the regions selected for the EDX analysis.

Following explantation, but prior to the hydrofluoric acid treatment, it was possible to observe an undulating and clump-like surface with cracks for all the implants (Figure 7, left column). In the EDX analysis, this surface contained above all magnesium, carbon, oxygen, phosphorus and calcium (Figure8(a)). It was also possible to find small amounts of sodium, potassium and neodymium. In the 13 and, even more so, in the 26 weeks groups, it was possible to observe crystalline, brittle surface structures which exhibited high amounts of calcium and phosphorus (Figure 8(b)).

**Figure 9:**
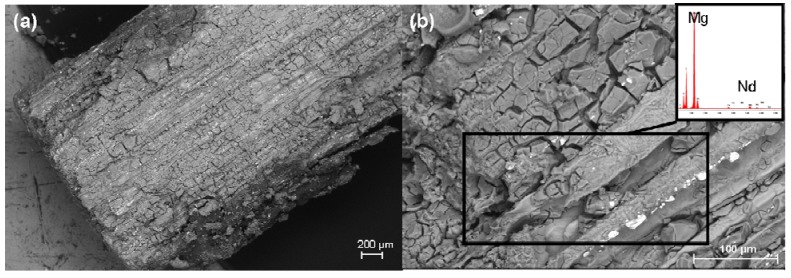
SEM images of the pin’s surface following hydrofluoric acid treatment, using a 26 weeks implant: (**a**) Distinct longitudinal grooves and clumpy structures; (**b**) EDX analysis: magnesium and neodymium in the grooves and clumpy structures

Following the hydrofluoric acid treatment (Figure 7, right column), implants from all the time groups exhibited different degrees of longitudinal grooves. In the 4 weeks groups, no changes in the cylindrical shape could be seen. In the 8 weeks groups, one implant exhibited eroded ends and an uneven surface. In this case, it involved the implant which was also noticeable in the stereomicroscope (however, not the implant which was radiologically more proximally situated in the tibia). The remaining implants retained their approximate cylindrical shape after 8 weeks as well as after 13 weeks. In the 26 weeks groups, two implants demonstrated eroded and jagged ends and corrosion pitting at several locations (Figure 7 (d) right column). It was possible to observe, particularly after 26 weeks, distinct grooves in the longitudinal direction, an uneven bumpy surface as well as clumps. In the EDX analysis, neodymium was partly found in the longitudinal grooves (Figure 9).

### 2.7. Determination of the Weight

**Figure 10:**
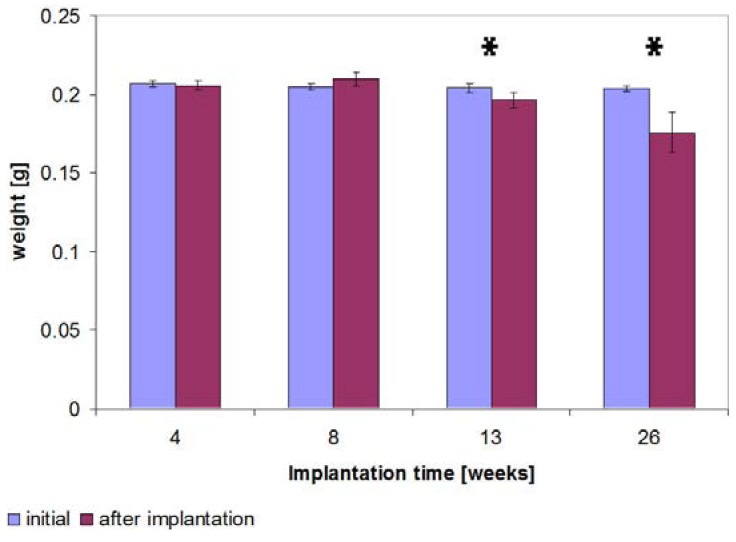
Implants’ weight measurements following hydrofluoric acid treatment for the different implantation times 4, 8, 13 and 26 weeks, significant weight reductions (p ≤ 0.05) in comparison to the initial values are verifiable from the 13th week and are identified by *.

Subsequent to explantation and to hydrofluoric acid treatment, the implants’ weights for the 4 and 8 weeks groups were not significantly changed in comparison to the initial weight of 0.21 g (SD 0.002), but a slight weight gain after 8 weeks of 0.005 g could be found. In contrast to this, after 13 weeks the weight significantly (p = 0.035) reduced by 3.7% in comparison to the initial weight of 0.20 g (SD 0.005) and was significantly (p = 0.01) lower than the weight after eight weeks 0.21 g (SD 0.004). After 26 weeks, the weight loss in comparison to the initial weight increased by 13.7% (to 0.18 g (SD 0.013)) (Figure 10) and was thus significantly (p = 0.05) higher than that after 13 weeks.

### 2.8. Results of the Three Point Bending

**Figure 11:**
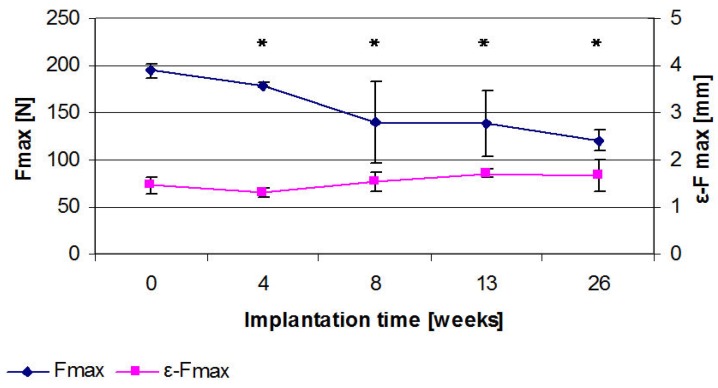
Three point bending tests for different implantation times after 4, 8, 13 and 26 weeks, the maximum force (Fmax) shows a distinct drop over the course of time but no significant change in elongation at fracture (ε-Fmax) (significant changes (p ≤ 0.05) in comparison to the initial value are identified by *).

For the initial implants, the maximum force (Fmax) was 194.5 N (SD 7.3) and the displacement (ε-Fmax) was 1.5 mm (SD 0.2). A distinct drop in Fmax was exhibited for all points in time following the respective implantation period (Figure 11). After 4 weeks, Fmax was reduced by 7.2% to 179.0 N (SD 2.7) (p = 0.002), after 8 weeks by 28.3% to 139.5 N (SD 43.5) (p = 0.026) and after 13 weeks by 28.9% to 138.2 N (SD 34.9) (p = 0.01). After 26 weeks, a loss of 38.3% (to 120.1 N (SD 10.8)) of the initial force was observed (p < 0.001). In all time groups, the ε-Fmax did not significantly change with respect to the initial value but showed a trend of increasing values. After 4 weeks, the ε-Fmax was 1.3 mm (SD 0.1) (p = 0.203), after 8 weeks 1.52 mm (SD 0.2) (p = 0.623), after 13 weeks 1.72 mm (SD 0.09) (p = 0.099), and after 26 weeks 1.7 mm (SD 0.3) (p = 0.108). 

### 2.9. Discussion 

Magnesium alloys are the object of current research for applications as degradable osteosynthetic materials [2,7,8].

The alloy LAE442 has been rated as suitable for this purpose [6,7]. However, the rare earths contained in this alloy are not reproducible with respect to the constitution of the individual elements [8]. For this reason, an alloy which contains the individual element neodymium instead of the rare earth mixture is to be investigated regarding its degradation behavior within the scope of the current study. In order to draw conclusions about the degradation behavior, cylindrical pins were implanted into the medullary cavity of 18 rabbits’ tibiae where they remained for 4, 8, 13 and 26 weeks. The degradation was radiologically and µ-computed tomographically investigated by means of density and volumetric changes taking place during the implantation period. Finally, weight measurements, SEM investigations, EDX analyses as well as three point bending tests were performed (following explantation and compared to the initial conditions).

During the investigation period, the pin’s radiological changes were observed as small, optical structural losses from, on average, the 12th week. In contrast to this, by using µCT investigations it was possible to verify the implants’ volume and density changes already from the 8th week. Buchmann describes in his book [28] that conventional X-ray images can still transform a weakening difference of 3% into an image which is discernible as a contrast. However, using computed tomography, weakening differences in tissue down to 0.1% can be discerned. This demonstrates the higher sensitivity of the CT evaluations. In the µCT, the density computations of each scan are actually performed using over 1,000 individual images by means of the µCT’s own software. This contrasts with the purely optical evaluation of the X-ray images in two planes. Moreover, depending on the position in the two-dimensional X-ray image, overlapping readily occurs which could influence the evaluation. For this reason, *in vivo* µCT investigations are superior to the radiological 2D images. Hühnerschulte *et al.* also state that the µCT investigations, due to their higher resolution, are better suited for evaluating bone reactions to the implant material than are x-ray images [29].

On the whole, the implants’ corrosion proceeded quite uniformly in the 1st week but, from the 12th week up to the end of the investigation period, was more irregular. This is demonstrated by the increasingly larger standard deviations in the µ-computed tomographic computations of the volume. 

In the current study, the LANd442 implants’ *in vivo* degradation behavior is mainly characterized by pitting corrosion. This is reflected, on the one hand, in the decrease of the average 3D-thickness, which is detected by *in vivo* µ-computed tomography, with increasing variances in all the test groups. These increases are also interpreted by other authors as pitting corrosion [29]. On the other hand, this is revealed in the SEM and stereomicroscopy examinations of the explanted pins after 26 weeks and thereby corresponds with the results from the *in vitro* tests [30]. These tests have established pitting corrosion in LANd442 implants using µ-computed tomography after incubating in SBF [30]. Pitting corrosion is also described by other authors for other magnesium alloys both *in vitro* [31,32] as well as *in vivo* in rabbit tibia [29,33]. It was possible for Krause *et al.* [33] to establish that no pitting corrosion occurred in LAE442 magnesium alloy after three and six months’ implantation in the rabbit’s tibia. Only Witte *et al*. describe partial, distinct pitting corrosion during the investigation of LAE442 pins in guinea pig’s femurs over an investigation period of up to 18 weeks [31]. However in this comparison, it must be considered that Krause *et al.* investigated extruded implants in their studies and, in contrast, Witte *et al.* employed gravity casted implants [31].

Influences on the corrosion can be induced by, on the one hand, the implant’s localization [34], probable damage of the pins [35,36] or, on the other hand, by microstructural differences such as, for example, the distribution of individual elements within the pins [20,29]. Apart from one pin, the implants’ correct positioning in this study, together with their sound condition and the rare earth neodymium’s imperfect homogeneous distribution on the implant’s surface, found in the SEM analysis, (Figure 8 and Figure 11 in the results section) allows one to assume that the current different corrosion rates can be attributed to individual differences in the rabbits or to different distributions of the implant’s individual elements. 

In order to more precisely characterize the pins prior to implantation for future research, additional SEM/EDX investigations could be carried out on transversally and longitudinally prepared sections and performing metallographic examinations of the microstructures as well as high-resolution µCT investigations. However, it is inhibitive that the investigation of the prepared sections depends on an implant’s destruction and thereby leads to implant loss. In contrast to this, high-resolution µCT investigations are non-destructive but only permit statements about the homogeneity and not about the elements’ distributions. 

In the current study, the implants’ low decreases in volume, expressed as a percentage, (5.5% after six months) appear not to initially correlate with the high losses in weight (14% after six months). A possible explanation for this divergence is the type of corrosion already described. The pitting corrosion takes place locally with shallow cavities, extend outwards and thus can lead to the excavation of smaller particles [32,37]. These particles, which are still only weakly attached to the implant, could remain unnoticed in the bone’s medullar cavity during explantation, or have been dissolved away by the hydrofluoric acid treatment, so that the determined weight is lower than one would expect based on the µ-computed tomographical calculations of the volumes. Another explanation for the difference between the calculated volume loss and the actual weight loss can be sought in the µCT evaluation. Here, an erroneous specification of the threshold value could occur by the experimenter [29,38]. However, in this study, the average volumes determined in the initial scans hardly deviate from the calculated volume (122.75 mm^3^) and the initially specified threshold was retained for the entire investigation period. Furthermore, the implant’s density loss occurring over the course of time can explain the pronounced weight loss in comparison to the volume loss computed in the µCT [29].

LANd442 loses volume and weight less quickly in comparison to LAE442, whose losses in volume and weight during the observation periods of three and six months are 14.9% and 22.4%, and 14.7% and 24.67%, respectively [6].Here however, the various evaluation methods of the volume loss must be considered. The volume loss of LAE442 was computed using the displacement method subsequent to removing organic material by means of treating in hydrofluoric acid [6,33]. In contrast to this, the volume loss of LANd442 was computed by means of *in vivo* µCT investigations, in which the corrosion products have possibly been included in the calculation, and could therefore account for the lower volume loss of LANd442 compared to LAE442. 

The corrosion products could also explain the slightly increased weight after 8 weeks (plus 0.005 g). *Ex vivo* studies also showed an increase of weight after implantation in the bovine udder model of pure magnesium pins and explain this by adherent corrosion products combined with a not yet pronounced degradation of the implant [39]. A similar effect could be possible *in vivo* and the 5 minutes immersion in hydrofluoric acid performed in this study could have been too short to thoroughly remove all corrosion products or organic material tightly attached to the implant. 

A rise in the computationally determined corrosion rate for LANd442 from 0.01 mm/y after 4 weeks to 0.02 mm/y after 8 weeks and to 0.03 mm/y after 12 weeks could be seen. The alloy attained its highest corrosion rate of 0.072 mm/y after 26 weeks. These differences in the computed corrosion values show that LANd442’s corrosion rate significantly increases with time. In contrast to this, Witte *et al.* [40] describes a decrease in the *in vivo* corrosion rate in the first 12 weeks. Similarly, LAE442’s computationally determined corrosion rate of 0.31 mm/y (SD 0.06) [40] after 12 weeks is much higher than that of LANd442 in this study.

The corrosion rates of LANd442 determined by Seitz *et al.* [30] lie significantly above these (2.6 to 15 mm/y) and exhibit complete degradation after 15 days. Thus, it is clear that the *in vitro* corrosion values in physiological media are not comparable with the *in vivo* corrosion rates. This is also confirmed by other authors for other magnesium alloys [41]. Furthermore, the work by Zhang *et al.* 2010 [41]. shows that the calculation of the annual corrosion rate can vary depending on the investigation time and the computationally determined annual corrosion rates from the literature are to be treated with reservation. 

In addition to this, the *in vivo* corrosion in the current work leads to density changes in the pins during the implantation period. It shows a small initial rise in the implant’s density in two of the four test groups from the second to the 4th week. This could be due to a deposit of calcium phosphate which, with 3.14 g/cm^3^ [42] possesses a higher density than magnesium (1.74 g/cm^3^) [42]. In this study, calcium and phosphorus was found on the implant surface of all the test groups with the aid of EDX analyses. *In vitro* studies in SBF describe that calcium and phosphorus rich apatite as well as amorphous phosphate, containing magnesium, precipitates onto the magnesium alloy’s surface [43,44,45]. This could also be the case *in vivo* and corresponds with the data in the literature [8]. Consequently, it could have been incorporated into the calculation of the pin’s density. 

The verified density loss can be due to the implant’s microstructural composition. Consequently, regions possessing high densities such as, for example, neodymium (6.99 g/cm^3^ [42]) or aluminum (2.7 g/cm^3^ [42]) could be increasingly decomposed whilst elements possessing lower densities, such as magnesium (1.74 g/cm^3^ [42]) or lithium (0.53 g/cm^3^ [42]) remain. Other authors also describe a change in the alloy’s composition by means of the degradation process as well as by the elimination of individual ions at different rates [29,45]. Moreover, the various phases within the magnesium alloy could exert an influence on the density since the α-phase sometimes corrodes more rapidly than the β-phase [37]. Furthermore, decomposed material could be replaced by endogenous elements possessing a lower density such as, for example, potassium (0.85 g/cm^3^ [42]) sodium (0.97 g/cm^3^ [42]) calcium (1.55 g/cm^3^ [42]) or phosphorus (1.84 g/cm [42]) during the decomposition. In the SEM/EDX investigations, it was possible to verify high levels of calcium and phosphorus, particularly after 13 and 26 weeks, together with a small amount of sodium and potassium on the implant’s surface. This was also the case in other studies [33,46]. The high content of Ca and P, verified in the SEM/EDX investigations, could further indicate that new bone grows around the residual implant material with increasing implantation time. This corresponds with the data from the literature [21,34]. For example, Reifenrath *et al.* reports trabecular bone-implant contact in 35% of the assessed histological sections for LAE442 after six month implantation time [21]. In order to draw reliable conclusions about whether it actually involves bone at the implant interface of LANd442 or, however, only the adsorption of calcium and phosphorus, as it was described in other *in vivo* investigation [8,37], this must be clarified in subsequent investigations for the new alloy LANd442. 

Biomechanically, significantly more distinct changes in the implants’ strength were demonstrated over the time period than for the volume and density changes. In particular, strength losses were observed between the 4th and 8th week. On employing LANd442 for osteosynthesis, increased load would thus be passed over to the bone in this time period. During fracture healing, the newly formed chondrocytes are, depending on the loading, replaced by osteoblasts in the formed callus between the 4th and 8th week [47,48], but the osteoblasts can only tolerate low loading [49]. According to the type of fracture and age of the patient, fracture healing in humans lasts from one to four months [50]. In order to maintain a low load on the healing bone in this period, the implant should remain strong for at least up to the 12th week [2]. For this reason, the LANd442 alloy would not be optimally suitable for adequately protecting the fracture. However, the generally slow loss of strength over the implantation period is to be assessed as favorable so that stress-shielding which can occur for stiff implants might be avoided [3]. In comparison to LAE442, the initial stability of LANd442 is significantly lower (LAE442: 252.7 N [33] LANd442: 194.5 N), whereas LANd442, with only 28.9% loss after 3 months, does not lose its strength as quickly as LAE442 (strength loss 40% [33]. However, the point in time at which the strength loss of LAE442 begins within the first three months is not known since the first investigation of the mechanical strength described by Krause *et al.* [33] was performed after 3 months. Thus an assessment regarding the protection of the fracture remains complicated. During the course of further implantation time, the decrease in strength is lower; for LAE442 a further 7% strength loss between three and six months [33] similar to that for LANd442 (a further 10% decrease of the initial stability between three and six months). 

In this study, LANd442 demonstrated, on the whole, a more retarded degradation than LAE442. This is described in the literature as a criterion for an improved biocompatibility [7,13,15,23]. However, it was possible to establish irregular degradation behavior by means of the true 3D-thickness and the diameter’s variance of the LANd442 implants. In contrast to this, the decomposition behavior of LAE442 is described by Krause *et al.* [33] as homogeneous and without discernible cross-sectional changes. 

To summarize, LANd442 compared to LAE442 is to be considered less suitable regarding its applications in load bearing bones. However, for applications in lightly loaded bone, LANd442 appears to be a potentially feasible alloy. Grain refining or the additional alloying of one or more rare earths may also exert a favorable effect on the mechanical properties. This is because the mixture of rare earths in LAE442 appears to have a distinct influence on the strength and the degradation behavior; after all, the alloy LACer442, which contained only cerium as an individual rare earth, was not comparable with the LAE442 alloy either [23].

Regarding the possibly toxic elements such as aluminum, a study by Yuen *et al.* evaluates that aluminum containing alloys should have no significant health risks but more caution is needed for multiple usage of such material [51]. For other elements like the rare earths NOEL (No Observed Effect Levels) are not given [51]. As LANd 442 has a low corrosion rate the authors of this study presume that no toxic effects due to degradation of the implants will arise, but in order to provide a conclusive assessment for applying LANd442 as an osteosynthetic material, *in vivo* studies regarding biocompatibility must be awaited. 

## 3. Experimental Section 

### 3.1. Manufacturing and Testing of the Implants

The magnesium alloy LANd442 is not commercially available and was specially manufactured for the present investigation. The nomenclature follows the ASTM Standard B275-90 [52]. Besides the main portion of magnesium, LANd442 contains 4 wt.% lithium, 4 wt.% aluminum and 2 wt.% neodymium. The alloy was manufactured using the casting process. Owing to the high reactivity of liquid magnesium, the material was melted in a shielding-gas atmosphere of argon within a closed system using a pressure of 0.2 bar. A foundry alloy containing 40% neodymium and 60% pure magnesium was mixed with aluminum and pure magnesium and melted at 760 °C. Lithium was subsequently added so that the corresponding target ratios were generated. After stirring for twenty minutes, the alloy was cast into a heated (500 °C) 130 mm diameter steel die coated with boron nitride. The ingot was then turned down to 120 mm diameter on a lathe. The material diameter was reduced from 120 mm to 30 mm by means of direct extruding at 350 °C. The final implant’s size, 2.5 mm diameter and 25 mm long, was obtained by turning the material. A total of 41 LANd442 implants were manufactured and used for this study. 

Prior to implantation for the *in vivo* investigations, the individual implants were weighed, washed using acetone and distilled water, packed in special sterilization bags and subsequently sterilized using gamma-radiation (27.1 kGy; BBF Sterilisationsservice, Kernen, Germany).

Prior to beginning the tests, the initial surface shape and structure of five exemplary pins were investigated by using a scanning electron microscope. 

Likewise, as an example, one implant was investigated using a reflection Theta-Theta diffractometer by means of a secondary graphite-monochromator (Stoe, Darmstadt, Germany) for generating CuK_α_ radiation. This was carried out in order to obtain the base values of the implants surface’s crystal structure for a subsequent comparison following explantation. The measurement was performed within the range from 5 to 70° 2θ using a step width of 0.01° 2θ with 1.5 seconds per step.

The implant’s mechanical properties were determined via three-point bending tests with a universal test machine (Zwick, Ulm, Germany) using five implants directly after their manufacture. For these tests, the implants were centrally located on two supports having a span of 15 mm and the bending punch was centrally placed between the two supports. Prior to the actual measurements, a preload of 2.5 N was set as a reference condition. The bending punch was subsequently traversed down at a constant rate of 1 mm per minute. The degree of deformation was measured using a displacement transducer. A sudden loss of 10% of the applied force was defined as the fracture criterion [7]. The measurements were concluded on attaining the fracture criterion or a bending punch displacement of 5 mm. The maximum force (Fmax [N]) and the specimen deflection at fracture (ε-Fmax [mm]) were recorded. 

### 3.2. *In Vivo* Investigations

#### 3.2.1. Test Animals

The animal tests were approved by the local government of Hanover according to paragraph 8 of the animal protection law under the reference number 33.9-42502-04-07/1363.

In total, 18 adult New Zealand White Rabbits (Charles River, Kisslegg, Germany) having a body weight of over 3 kg were included in the investigations. 

#### 3.2.2. Surgical Procedure

As described by Thomann *et al.* [7], the implants were introduced under general anaesthetic into the central medullary cavity via an entrance medially drilled into the tibial plateau. To induce anaesthesia, s-ketamine hydrochloride (10 mg/kg, CP-Pharma, Burgdorf, Germany) and medetomidine (0.125 mg/kg, Domitor^®^, Pfizer GmbH, Berlin, Germany) were intramuscularly injected. For maintaining anaesthesia 2 to 3 vol% Isofluran (Isoba^®^, Essex Pharma GmbH, Munich, Germany) was employed. The wound was closed using resorbable suture material (SAFIL^®^ violet 4/0, B. Braun Melsungen AG, Melsungen, Germany). Pain was treated using Meloxicam (subcutically, 0.15 mg/kg, Metacam^®^, Boehringer Ingelheim, Ingelheim, Germany) and intra procedurally using Fentanyl dihydrogencitrate (10 µg/kg, Fentanyl-Janssen^®^, Janssen-Cilag GmbH, Neuss, Germany). Enrofloxacine (10 mg/kg, Baytril^®^2.5%, Bayer HealthCare, Leverkusen, Germany) was employed as a prophylactic antibiotic. 

Meloxicam and Enrofloxacine were administered post operatively for a further ten days at the specified dosage. 

#### 3.2.3. Post-Operative Investigation Period

In total, 36 implants were implanted over 4 (n = 10), 8 (n = 10), 13 (n = 6) and 26 (n = 10) weeks. The animals were clinically examined daily. 

#### 3.2.4. Radiological Investigation

**Figure 12:**
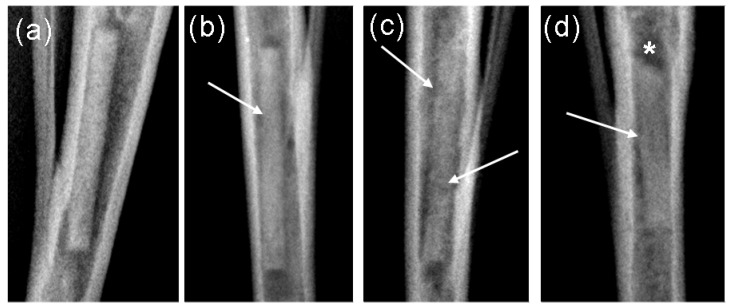
Assessment of the implant’s degradation by means of anterior-posterior x-ray images: (**a**) Score 0: as on the day of implantation; (**b**) Score 1: low structural loss without shape change; (**c**) Score 2: marked structural loss without shape change; (**d**) Score 3; significant structural loss with shape change. Arrows indicate the structural loss, stars indicate shape changes.

Post operatively, the location of the implants was radiologically controlled. Digital x-ray images (Practix 160, Philips, Hamburg, Germany) of the tibiae were recorded weekly in the mediolateral and anterior-posterior beam path using 48 kV and 6.3 mAs. By means of the x-ray images, the appearance of the implants were assessed using semi-quantative scores [23] from 0 (no change), 1 (low structural loss of the implant without shape change), 2 (marked structural loss of the implant without shape change) and 3 (significant structural loss with shape change of the implant). Besides this, the occurrence of gas (low to high levels) was assessed on a computer (dicomPACS^®^ vet Version 5.2.4; Oehm und Rehbein GmbH, Rostock, Germany) (Figure 12).

#### 3.2.5. *In Vivo* µ-Computed Tomography

*In vivo* µ-computed tomographic (µCT) images (XtremeCT, Scanco Medical, Zürich, Switzerland) were regularly recorded to exactly determine the intra and inter individual degradation of the implants. For up to 8 weeks, the µCT-investigations were performed every 14 days and then every 4 weeks as well as directly following the animal’s euthanasia.

The *in vivo* µCT images were performed under anaesthesia similar to the previously described operation. Following intubation, the rabbits were laid in the dorsal position in a frame specifically made for the µCT investigation. 

Topograms of the rear limbs were produced, in which the implants could be clearly discerned, and the region to be examined was established from approximately 0.3 cm below the implant’s distal end up to the knee joint space. The animals were scanned using a resolution of 41 µm at 60 kV and 900 µA and an integration time of 100 ms. The implants were subsequently manually outlined and measured by means of the software µCT evaluation program V6.1 (XtremeCT, Fa. Scanco Medical, Zürich, Switzerland) with a threshold value of 138.

For each tomogram, 1,200–1,700 individual images were generated during an investigation period of 22–30 minutes. Each individual image consisted of 1,000 projections between 0° and 180°. 

In order to describe more precisely the degradation behavior, the true 3D-thickness was computed. During this computation, the implant is virtually filled with balls of a maximum diameter and then the average ball size as well as the standard deviation of the implant’s ball sizes (subsequently designated as variance) is computed [29]. A high variance therefore arises for various diameters within an implant and this is an indication of a non-uniform surface structure (e.g., pitting corrosion) [29].

The implant’s corrosion rate was calculated using the *in vivo* µCT data for a time period of 26 weeks according to the following formula according to Witte *et al.* [40]:

CR = ΔV/(A × t)
(1)

here, CR [mm/year] is the corrosion rate, ΔV [mm^3^] the volume loss, A [mm^2^] the area which was subjected to the corrosion and t [days] the implantation period.

### 3.3. Investigation of the Implants Following Removal

At the end of their respective observation period, the animals were anaesthetized using s-ketamine hydrochloride (intra muscularly, 20 mg/kg, CP-Pharma, Burgdorf, Germany) and xylazinhydrochloride (intra muscularly, 5 mg/kg, Serumwerk Berburg AG, Berburg, Germany) and painlessly euthanised by means of an intracardiac injection of Pentobarbital (230 mg/kg, Narkodorm^®^ CP-Pharma, Burgdorf, Germany). After opening the left tibiae using a Dremel rotary cutter, the implants were carefully removed.

#### 3.3.1. X-Ray Diffractometery

Using an 8 week implant as an example, the composition (analysis of the crystal structure) of the implant’s surface, including the corrosion layer, was investigated by means of a reflection Theta-Theta-diffractometer immediately after explantation. The measurement was compared with the specimen, which was previously described (see material and methods: manufacturing and testing of the implants), of the same alloy after its manufacture. 

#### 3.3.2. Investigation of Implants Using Stereomicroscopy

The implants’ surface was investigated and descriptively evaluated using a stereomicroscope (MZ8, Leica, Solms, Germany) immediately after explantation and after treating in hydrofluoric acid. Images of the implant’s surface were produced using ×2.0 magnification. 

#### 3.3.3. Scanning Electron Microscopy and Energy Dispersive X-Ray Spectroscopy

Scanning electron microscopic (SEM) examinations of the implant’s surfaces were carried out after explantation as well as after treating in hydrofluoric acid. An element analysis was performed at selected locations using energy dispersive X-ray spectroscopy (EDX) (LEO 1455VP, Zeiss, Oberkochen, Germany and EDAX Genesis, EDAX, Mahwah, USA). The SEM images of the implants were recorded using EBSD (electron backscatter diffraction) and VPSE (variable pressure secondary electrons) methods with a resolution of 5 nm. These were evaluated descriptively. 

#### 3.3.4. Investigation of the Implants after Treating in Hydrofluoric Acid

Subsequent to the investigations mentioned above, the implants were treated in an immersion bath of hydrofluoric acid (40%) for 5 minutes in order to remove attached tissue and the corrosion layer [53,54,55]. Following this, the implants were washed with distilled water and ethanol for 10 seconds each and dried in air. 

Subsequent to the hydrofluoric acid treatment, the mechanical properties of all the explanted pins were determined analogous to the mechanical testing (three-point bending) in the initial condition. 

#### 3.3.5. Determination of the Weight

The implant’s weight was determined using precision scales (AB204-S/Fact, Metler Toledo, Giessen, Germany) prior to the tests as well as after the hydrofluoric acid treatment. The weight loss data is expressed as a difference in percent of the remaining implant to the initial weight prior to implantation.

### 3.4. Statistics

The statistical analysis was carried out using Microsoft Office Excel^®^, version 2003 and SPSS^®^, version 17.0. The mean values and the standard deviations were computed for the various time groups. After testing for a normal distribution, statistical differences for the implant’s volume, the true 3D-thickness, including their variances, and the implant’s weight were determined as a function of time by means of the T-test for paired samples. The density change was statistically investigated using tests according to Wilcoxon. The data from the three-point bending tests were analyzed using the T-test for independent samples with respect to significant differences between the implantation periods. Comparisons between the groups were performed using a univariant variance analysis with subsequent post-hoc tests (Tukey or Games-Howell).

Values of p ≤ 0.05 and p ≤ 0.01 were rated as significant and highly significant, respectively. 

## 4. Conclusions

In summary the new magnesium based alloy LANd442 has an increasing corrosion rate over time which is favorable for biodegradable osteosynthesis materials especially in order to possibly prevent the so-called “stress shielding”. Furthermore, the slow degradation rate of LANd442 and its mostly homogeneous degradation behavior are advantageous and thus LANd442 may be a suitable osteosynthesis material for mechanically less demanding applications. However, LANd442 has to be considered as less suitable for the use in osteosynthesis of load bearing bones than LAE442, due to its lesser initial strength.
